# Nevoid Hyperkeratosis of the Nipple and Areola: A Case Report

**DOI:** 10.7759/cureus.91504

**Published:** 2025-09-02

**Authors:** Diana Morales-Olvera, Nadia Aranza Mota Díaz, Elisa Vega-Memije, Miren Lorea Cárdenas Hernández, Ana L Ramirez Teran

**Affiliations:** 1 Department of Dermatologic Surgery, Hospital General "Dr. Manuel Gea González", Mexico City, MEX; 2 Faculty of Medicine, Universidad Nacional Autónoma de México (UNAM), Mexico City, MEX; 3 Department of Dermatology, Hospital General "Dr. Manuel Gea González", Mexico City, MEX; 4 Department of Dermatopathology, Hospital General "Dr. Manuel Gea González", Mexico City, MEX

**Keywords:** areola hyperkeratosis, nevoid hyperkeratosis, nipple, tretinoin, verrucous dermatoses

## Abstract

We present a case of nevoid hyperkeratosis of the nipple and areola (NHNA) with emphasis on its clinical, dermoscopic, and histopathological features and the diagnostic challenges it poses. NHNA is a rare, generally benign dermatologic condition characterized by verrucous, hyperpigmented lesions of the nipple-areola complex. We report the case of a 21-year-old woman presenting with bilateral, symmetrical areolar thickening of progressive onset and no associated symptoms. A clinical diagnosis was made, and 20% urea treatment was prescribed, with no clinical improvement. One year later, due to lesion progression, a skin biopsy was performed to confirm the diagnosis histopathologically; 0.05% topical tretinoin was initiated, but the patient was lost to follow-up. This case highlights the importance of differential diagnosis and long-term follow-up in uncommon dermatoses of the nipple-areola complex.

## Introduction

Nevoid hyperkeratosis of the nipple and areola (NHNA) is a rare, benign dermatologic condition characterized by verrucous, hyperpigmented plaques affecting the nipple-areola complex. First described in 1923, fewer than 200 cases have been reported in the literature to date [1]. Although its exact pathogenesis remains unclear, hormonal influences have been proposed due to its predominance in women of reproductive age [2]. While typically asymptomatic, NHNA may raise aesthetic concerns that lead patients to seek dermatologic consultation [3].

Several classification systems have been proposed to better define NHNA. In 1938, Levy-Franckel described three types: (1) the extension of an epidermal nevus; (2) association with other dermatoses, such as ichthyosis, acanthosis nigricans, or cutaneous lymphomas, usually bilateral in presentation; and (3) idiopathic forms [4]. More recent classifications group NHNA into two main categories: primary (idiopathic) and secondary (associated with other conditions such as epidermal or organoid nevi, leiomyomas, verruca vulgaris, or seborrheic keratoses) [5].

NHNA tends to persist indefinitely [5]. Although various treatment modalities have been described, no standardized or consistently effective therapy has been established [1,5,6].

We present a case of bilateral, symmetrical NHNA in a young woman, emphasizing the clinical, dermoscopic, and histopathological findings, as well as the therapeutic approach and its limitations.

## Case presentation

**Figure 1:**
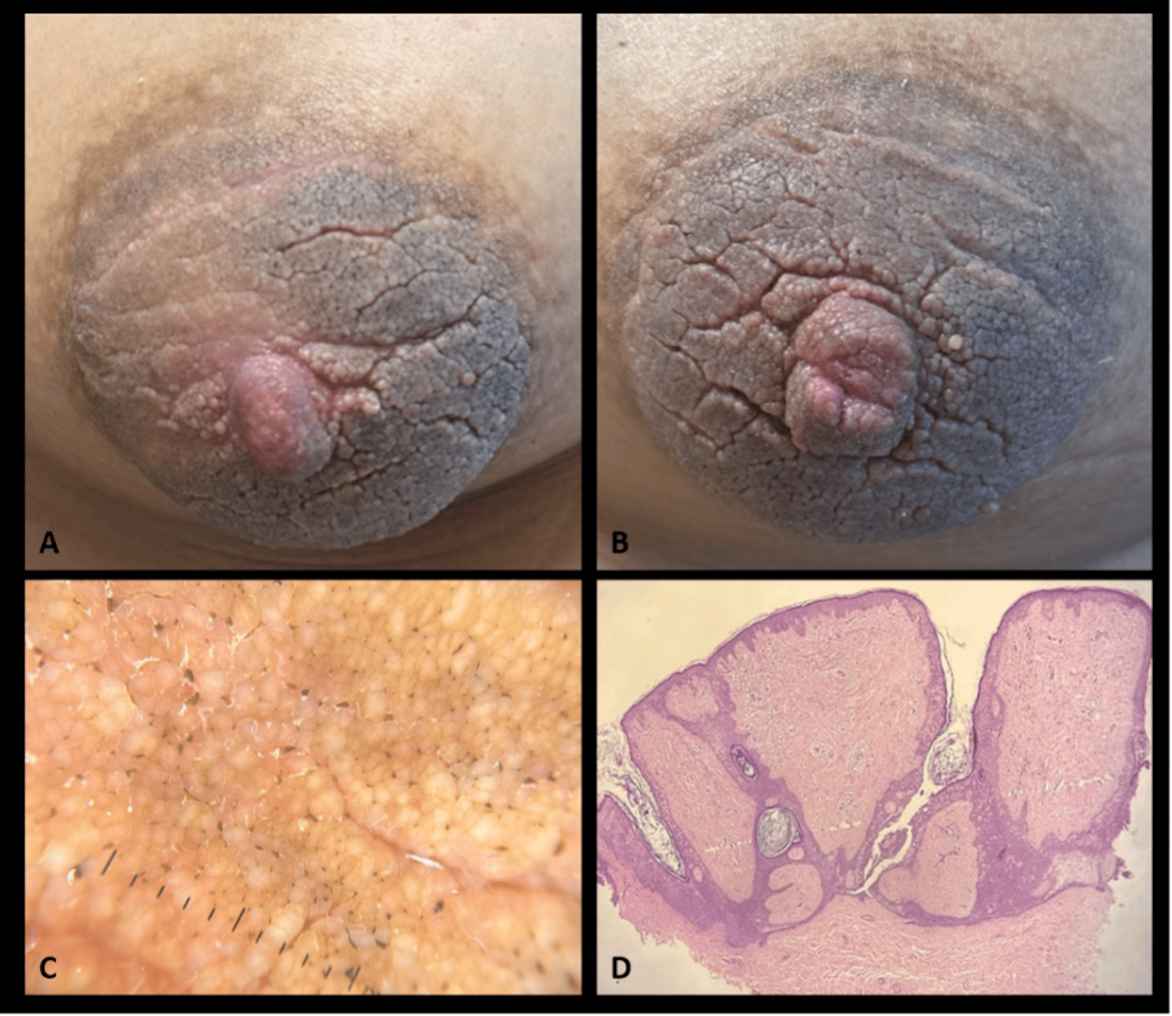
Clinical, dermoscopic, and histopathological images (A) Right areola and (B) left Areola. Well-demarcated, oval, grayish-brown verrucous plaques with a cerebriform appearance. (C) Polarized dermoscopy with ridges and fissures, associated with openings such as comedones and alternating with well-defined yellow areas. (D) Histopathological microphotograph (hematoxylin and eosin, original magnification of 10×) showing focal hyperkeratosis, acanthosis, and fused and elongated interpapillary processes intersecting to form a trabecular-like net with storiform-patterned fibrous stroma

A 21-year-old woman was referred from the gynecology service due to the progressive, bilateral, and symmetrical thickening of the nipples and areolas over several years. The only notable antecedent was a superficial blunt trauma to the breast two months prior to evaluation. The patient was asymptomatic, with no history of pregnancy or medication use. Her body mass index was 23 kg/m². Physical examination revealed a dermatosis affecting both breasts, involving the areola and nipple. The lesions consisted of well-demarcated, oval, grayish-brown verrucous plaques measuring approximately 8×7 cm, with a cerebriform appearance (Figure 1A, 1B). Polarized dermoscopy revealed ridges and fissures associated with openings resembling comedones, alternating with well-defined yellow areas (Figure 1C). No abnormalities were noted in the remainder of the breast examination. There were no signs of acanthosis nigricans or epidermal nevi elsewhere.

A clinical diagnosis of nevoid hyperkeratosis of the nipple and areola was established. Given the benign and asymptomatic nature of the condition, the patient initially declined a skin biopsy for histopathological confirmation. Treatment with 20% topical urea once daily was prescribed, but no clinical improvement was observed.

One year later, the patient returned with concerns about aesthetic appearance and lesion progression, prompting a skin biopsy. Histopathological findings revealed basket-weave hyperkeratosis with focal orthokeratosis, acanthosis, and mild papillomatosis. Interpapillary projections were elongated and fused intersecting to form a trabecular-like net with a stroma exhibiting a storiform and fibrous pattern. The papillary dermis showed mild fibrosis and a superficial perivascular lymphocytic infiltrate (Figure 1D).

Clinical and histopathological findings confirmed the diagnosis of nevoid hyperkeratosis of the nipple and areola. The patient was treated with topical tretinoin 0.05% cream nightly for three months, but the patient was lost to follow-up.

## Discussion

Based on the most recent classification, our case represents an idiopathic presentation of NHNA, with no associated systemic or dermatologic conditions.

Although NHNA can affect both sexes, 80% of reported cases occur in women, primarily during the second and third decades of life [4,7].

The pathogenesis remains unclear, but a hormonal theory is widely accepted, supported by the condition’s appearance during puberty, exacerbation during pregnancy, and occurrence in men receiving estrogen therapy [3,5]. Certain medications, such as spironolactone and vemurafenib, have also been implicated [8,9].

Clinically, the condition presents as unilateral or bilateral brown verrucous plaques localized to the nipple and/or areola [1]. Yellowish discoloration or desquamation may also be observed in some cases [6,10]. Dermoscopy reveals a papillomatous surface with pink homogeneous areas, whitish desquamation, and brown hyperkeratotic deposits within ridges [11,12].

Histopathological examination reveals hyperkeratosis, acanthosis, papillomatosis and keratin plugs, orthokeratosis, basal hyperpigmentation, and the elongation of interpapillary ridges [13,14]. A superficial perivascular lymphocytic infiltrate may be seen in the dermis, features that can also be observed in acanthosis nigricans, seborrheic keratosis, or epidermal nevi [5]. Accurate clinicopathologic correlation is essential for differentiation, as was performed in this case.

Although NHNA is primarily diagnosed through clinical and histopathological correlation, dermoscopy holds significant value, offering a novel approach that enhances both diagnostic precision and therapeutic decision-making [15].

Differential diagnosis includes Paget disease of the breast, superficial basal cell carcinoma, and Bowen’s disease, often presenting as paraneoplastic dermatoses [6,16]. In younger patients, the main differential diagnoses include verruca vulgaris, seborrheic keratosis, frictional hyperkeratosis, and hyperkeratosis associated with other dermatoses. Viral warts are particularly common in children and can appear anywhere on the skin surface, but they are readily distinguishable from NHNA by clinical and dermoscopic features. Seborrheic keratosis, while it may mimic NHNA, is rare on the nipple in young children and is typically multiple in older individuals and characterized by sharply demarcated papules. Careful clinical history and complete skin examination are essential to rule out these conditions [4,11].

First-line treatments include keratolytics such as lactic acid and salicylic acid, though their efficacy is often limited. Topical corticosteroids may relieve pruritus but have minimal effect on the lesion. Topical retinoids (e.g., tretinoin 0.05%), systemic retinoids (e.g., isotretinoin, etretinate, and acitretin), and topical vitamin D analogs (e.g., calcipotriol) have shown variable clinical outcomes [5].

In the management of NHNA, special attention must be given to restoring both the functional integrity and the aesthetic appearance of the breast, particularly in young patients. The CO₂ laser offers distinct advantages, including surgical precision, a bloodless field, and reduced postoperative discomfort, making it an effective therapeutic option that provides favorable cosmetic outcomes with minimal complications [17].

Cryotherapy with liquid nitrogen has shown favorable clinical outcomes. Surgical options include tangential excision, curettage, or complete areolar resection, followed by skin graft reconstruction [5].

Despite its benign nature, NHNA may lead to psychological distress due to aesthetic concerns and has been reported to interfere with breastfeeding [13,18]. Therefore, clear communication regarding prognosis and management options to the patient is essential.

## Conclusions

We present this case to raise awareness of this uncommon dermatosis among dermatologists and gynecologists. Although benign, NHNA should be considered a diagnosis of exclusion in the differential diagnosis of potentially aggressive nipple-areola lesions. Patient education and reassurance are fundamental, especially considering the absence of a universally effective treatment. Establishing a clinical registry with proper follow-up may also help monitor recurrence and guide further aesthetic management.
